# Genome-wide association study of berry-related traits in grape [*Vitis vinifera* L.] based on genotyping-by-sequencing markers

**DOI:** 10.1038/s41438-018-0089-z

**Published:** 2019-01-01

**Authors:** Da-Long Guo, Hui-Li Zhao, Qiong Li, Guo-Hai Zhang, Jian-Fu Jiang, Chong-Huai Liu, Yi-He Yu

**Affiliations:** 1 College of Forestry, Henan University of Science and Technology, Luoyang, 471023 Henan Province China; 2 Zhengzhou Fruit Research Institute, Chinese Academy of Agricultural Sciences, Zhengzhou, 450009 Henan Province China

**Keywords:** Natural variation in plants, Plant breeding, Natural variation in plants, Plant breeding

## Abstract

Deciphering the genetic control of grape berry traits is crucial for optimizing yield, fruit quality, and consumer acceptability. In this study, an association panel of 179 grape genotypes comprising a mixture of ancient cultivars, landraces, and modern varieties collected worldwide were genotyped with genotyping-by-sequencing using a genome-wide association approach based on 32,311 single-nucleotide polymorphism (SNP) markers. Genome-wide efficient mixed-model association was selected as the optimal statistical model based on the results of known control loci of grape berry color traits. Many of the associated SNPs identified in this study were in accordance with the previous QTL analyses using biparental mapping. The grape skin color locus was found to be associated with a *mybA* transcription factor on chromosome 2. Two strong and distinct association signals associated with berry development periods were found on chromosome 16. Most candidate genes of the interval were highlighted as receptor-like protein kinase. For berry weight, significant association loci were identified on chromosome 18, as previously known, and on chromosome 19 and chromosome 17, as newly mapped. Berry flesh texture was newly located on chromosome 16; candidate genes in the interval were related to calcium. Berry flavor was determined on chromosome 5. Genomic regions were further investigated to reveal candidate genes. In this work, we identified interesting genetic determinants of grape berry-related traits. The identification of the markers closely associated with these berry traits may be useful for grape molecular breeding.

## Grapes: The genetic basis of fruit traits

Genetic analysis of 179 types of vine has revealed regions of the genome and associated genes and proteins that control many of the key traits in the grapes. These traits are crucial for determining fruit quality and yield, in addition to conferring flavors that are highly valued by consumers and wine makers. Researchers in China, led by Da-Long Guo at Henan University of Science and Technology, used DNA sequencing to analyze genetic markers in a mixture of ancient, traditionally cultivated and modern grape varieties. The analysis identified specific regions of the genome linked to grape fruit development, weight, texture, skin color and flavor. The molecular basis of the effects of the identified genetic regions were also explored. The findings will help generate new varieties of grapes for the food and wine industries.

## Introduction

Grape [*Vitis vinifera* L.] is an economically important fruit-tree crop in many temperate and subtropical countries. Its economic importance and potential health benefits make it a common choice for fruit-tree cultivation. As a result of long-term natural selection and artificial domestication and breeding, many fruit variations have accumulated in grape, with wide diversities of berry color, size, weight, texture, aroma, and shape. These variations could serve as important resources for grape breeding and genetic improvement. A better understanding of their genetic control could facilitate the selection of desirable traits. Most fruit-related agronomic traits are complex quantitative traits. The efficiency of selecting desirable traits could be enhanced by gaining a better understanding of their genetic control.

The identification of genotype-to-phenotype associations is an important focus in plant breeding. The genetic control of major berry-related traits in grape, such as phenology, berry weight, berry firmness, and seedlessness, has been explored via simple sequence repeat (SSR) markers in biparental populations^1–6^. However, the results of quantitative trait locus (QTL) mapping usually vary greatly among genotypes^7^. A biparental population makes use only of the recent recombination information that arose during the crossing, which is often population specific and less applicable against wider genetic backgrounds^8^. In contrast, studies of diverse populations (natural populations) can exploit all historical recombination events accumulated in the sampled individuals^9^. The wide diversity in grape genotypes remains largely underexplored.

The advent of next-generation sequencing (NGS) has made it possible to obtain genome-wide coverage markers affordably using the “reduced representation approach” in any species. The flexibility and low cost of genotyping-by-sequencing (GBS) make it an excellent tool in genome-wide association studies (GWASs)^10,11^. Genome-wide association mapping is a widely used method to dissect the genetic basis of complex traits^9^. In a large plant natural population, GWAS was a powerful method for identifying QTLs with multiple alleles at the whole-genome level based on linkage disequilibrium (LD)^11,12^. In a recent study, a GWAS was performed with 129 peach accessions to identify QTLs controlling 12 key agronomic traits^13^. A GWAS was conducted to explore the genetic structure and domestication history of the grape collection of the United States Department of Agriculture (USDA)^14^. The genetic basis of leaf shape was investigated through the GWAS evaluation of 961 grape genotypes using Vitis9kSNP array^15^. Several other GWAS analyses have been conducted to investigate grape genetic diversity^16,17^, aroma^18^, and a few candidate genes^19^. However, the genetic mechanisms controlling the phenotypic diversity of grape berry traits based on GWASs have yet to be fully explored.

Population structure and kinship from the genetic background are two common indirect, non-causal associations that lead to false positives in GWASs. Various software packages have been developed to eliminate false positives in different situations. TASSEL (Trait Analysis by aSSociation, Evolution, and Linkage) employs general linear model and mixed linear model approaches to simultaneously account for population structure and unequal relatedness among individuals^20^. Efficient Mixed-Model Association eXpedited (EMMAX) assesses variance components (or their ratios) and then fixes them to test genetic markers^21^. Genome-wide Efficient Mixed-Model Association (GEMMA) was developed to assess population parameters for individual markers^22^. However, there is no general software package applicable to all association panels.

To improve our understanding of the genetic bases of grape berry agronomic traits, an association analysis of 179 grape elite genotypes from the primary core collection^23^ of the National Grape Germplasm Repository at Zhengzhou Fruit Research Institute of the Chinese Academy of Agricultural Sciences was conducted based on the GBS method. The primary objective of this study was to use a GWAS to identify single-nucleotide polymorphisms (SNPs) associated with important berry agronomic traits. The identified SNPs may be used to improve the fruit quality of grape.

## Materials and Methods

### Materials and phenotyping

For genome-wide genotyping, a total of 179 grape genotypes (Supplementary Table S1) were collected from the National Grape Germplasm Repository at Zhengzhou Fruit Research Institute of the Chinese Academy of Agricultural Sciences (113°42`E and 34°42`N). The annual precipitation of Zhengzhou in 2014 and 2015 was 551.6 and 689.1 millimeters, respectively, and the corresponding annual average temperature was 16.3 and 15.9 °C, respectively. Genotypes consisted of farmers’ landraces and released cultivars from different countries. Eight major grape berry-related traits were measured: berry development period (BDP: the period in days from flowering to physiological maturity); cluster size (CS: area of the fruit cluster, cm^2^); cluster density (CD: very loose, loose, medium, dense, very dense); berry weight (BW, g); berry flesh texture (BFT: soft, medium, slightly firm, very firm); berry color (BC: green–yellow, rose, red, gray, red–violet, blue–black); berry shape (BS: obloid, globose, broad ellipsoid, narrow ellipsoid, obovoid, finger-shaped); and berry flavor (BF: none, muscat, foxy, herbaceous, other). Ten ripe clusters were harvested from each plant and subjected to measurements of the above traits. Field experiments in which berry traits were conducted in 2014 and 2015. In general, three plants per variety were maintained. For quantitative traits (BDP, CS, and BW), the average phenotypic value of each trait and year was considered for the association tests^8^. Qualitative traits (CD, BFT, BC, BS, and BF) were verified in the second season, and no differences between years were observed. The equality of variance and means were analyzed by analysis of variance (ANOVA). Pearson correlation coefficients of traits between 2014 and 2015 were calculated. All analyses were conducted using R 4.3.2.

### DNA extraction, sequencing, and SNP calling

Young and fresh leaves were harvested from each individual grape genotype. Samples were immediately stored in liquid nitrogen and moved to a −80 °C freezer. DNA was extracted according to the method of Zyprian et al^24^. Restriction enzyme *ApeK* I was used for library preparation following the GBS protocol of Elshire et al^10^. GBS was conducted by HiSeq 2000 at the Beijing Genomics Institute (Shenzhen, People’s Republic of China). The raw sequencing data and SNP calling were analyzed using SOAP family software (http://soap.genomics.org.cn/). The short reads were aligned to the reference grape genome (http://genomes.cribi.unipd.it/DATA/GENOME_12×/) using SOAPaligner/soap2 (http://soap.genomics.org.cn/), and SOAPsnp v1.05 was used for SNP calling.

### LD analysis, population structure, and clustering

Calculations of pairwise LD, r^2^, between SNPs were based on SNPs within 1 Mb windows using PLINK v1.07 software^25^. The program ADMIXTURE was run 1000 times for K values 2–10 to generate admixture proportions. Then, the best value of K was determined by cross-validation (CV) and log-likelihood estimates. The log-likelihood difference between the minimum and maximum of each K was calculated. To construct the neighbor-joining tree, PHYLIP 3.696 software was used.

### Genome-wide association analysis

To ensure the accuracy of the results, SNPs with a missing genotype frequency greater than 0.05 or a minor allele frequency (MAF) less than 0.05 were excluded from analysis. Imputation was not performed. Four methods were used to implement the GWAS: (1) Plink:^25^ Given that none of the traits were binary and that the category phenotypes could be treated as special quantitative traits, the “–assoc” option was applied to implement the regression statistics and write the results to a.qassoc file. (2) TASSEL 5.0:^20^ The K+Q module (mixed linear model, MLM) was used in the TASSEL analysis. K was a kinship matrix built by TASSEL, and the best admixture results representing population membership served as covariates in the model, denoted Q. (3) EMMAX:^21^ On the command lines the options emmax-kin -v -h -s -d 10; emmax -v -d 10 were entered for the creation of the identity-by-state (IBS) relationship matrix and for the association test, respectively. (4) GEMMA:^22^ Association mapping was performed in R using the GEMMA implementation of the standard linear mixed model, y = Wα + Xβ + u + ε see Zhou et al^22^.

The final genome-wide significance thresholds were calculated by GEC^26^, which applied Bonferroni correction according to the effective number of independent SNPs. The Q–Q plots (Supplementary Figure S1) indicated that the GEMMA model fit the data well. The SNPs with −log10 (*P*) > 5 were considered significant. Manhattan plots of −log10 (*P*) values for each SNP versus chromosomal position were generated as the GEMMA results.

### Gene annotation

The regions upstream and downstream of the significant SNPs along the genome were investigated to identify the annotated genes by scanning the genome in ~1 Mb windows. In the case where two moderately distant SNPs showed association, the entire genome region between them was explored. The annotated gene sequences of the 12 × V2 grape genome assembly were retrieved from CRIBI (http://genomes.cribi.unipd.it/) to identify the target genes for the corresponding associated regions.

## Results

### Phenotypic data

Although most existing association software was designed for quantitative traits, quantitative response variables representing categorical data can be used to conduct association tests for qualitative traits^21^. Accordingly, the data for the CD, BFT, BC, BS, and BF traits were recorded as discrete data. The phenotypic characters of these qualitative traits were verified in the second season, and no difference between 2014 and 2015 was observed. The grape genotypes used in this study showed broad variation for three other traits: BDP, BW, and CS (Supplementary Table S1). The average phenotypic values of BDP, BW, and CS were 81.03 d (range 50–110 d), 3.93 g (range 0.85–11.92 g), and 144.86 cm^2^ (16.67-393.08 cm^2^), respectively. The ANOVA results indicated that these three traits were significantly influenced by genotype; however, year and genotype by year had no significant effects (Table 1). Pearson’s phenotypic correlation coefficients between the two years were highly significant for all three traits (*P* < 0.01) (Table 1). Most of the genotypes in the population performed similarly between the two years, as reflected by the insignificant genotype × year variance components. Therefore, the mean values of these three traits across the two years were used in the following analysis.

**Table 1:** Phenotypic variation and ANOVA results for the traits of BDP, BW, and CS in grape over 2 years

Trait	Year	Min	Max	Mean ± SD	Pearson correlation coefficient of years	P-value of ANOVA		
						Genotype	Year	Genotype × year
BDP	2014	50 d	110 d	80.49 ± 13.42 d	0.99^**^	0.047^*^	0.452	0.894
	2015	51 d	110 d	81.57 ± 13.69 d				
BW	2014	0.85 g	11.3 g	3.98 ± 2.10 g	0.95^**^	0.009^**^	0.650	0.987
	2015	0.92 g	11.92 g	3.88 ± 1.93 g				
CS	2014	16.67 cm^2^	393.08 cm^2^	143.97 ± 62.64 cm^2^	0.79^**^	8.08e-06^**^	0.773	0.983
	2015	21.36 cm^2^	351.4 cm^2^	145.76 ± 57.63 cm^2^				

*BDP* the period from flowering to physiological maturity, *BW* berry weight, *CS* cluster size

* and ** indicate significance at the *P* < 0.05 and *P* < 0.01 levels, respectively

### SNP calling

**Fig. 1:**
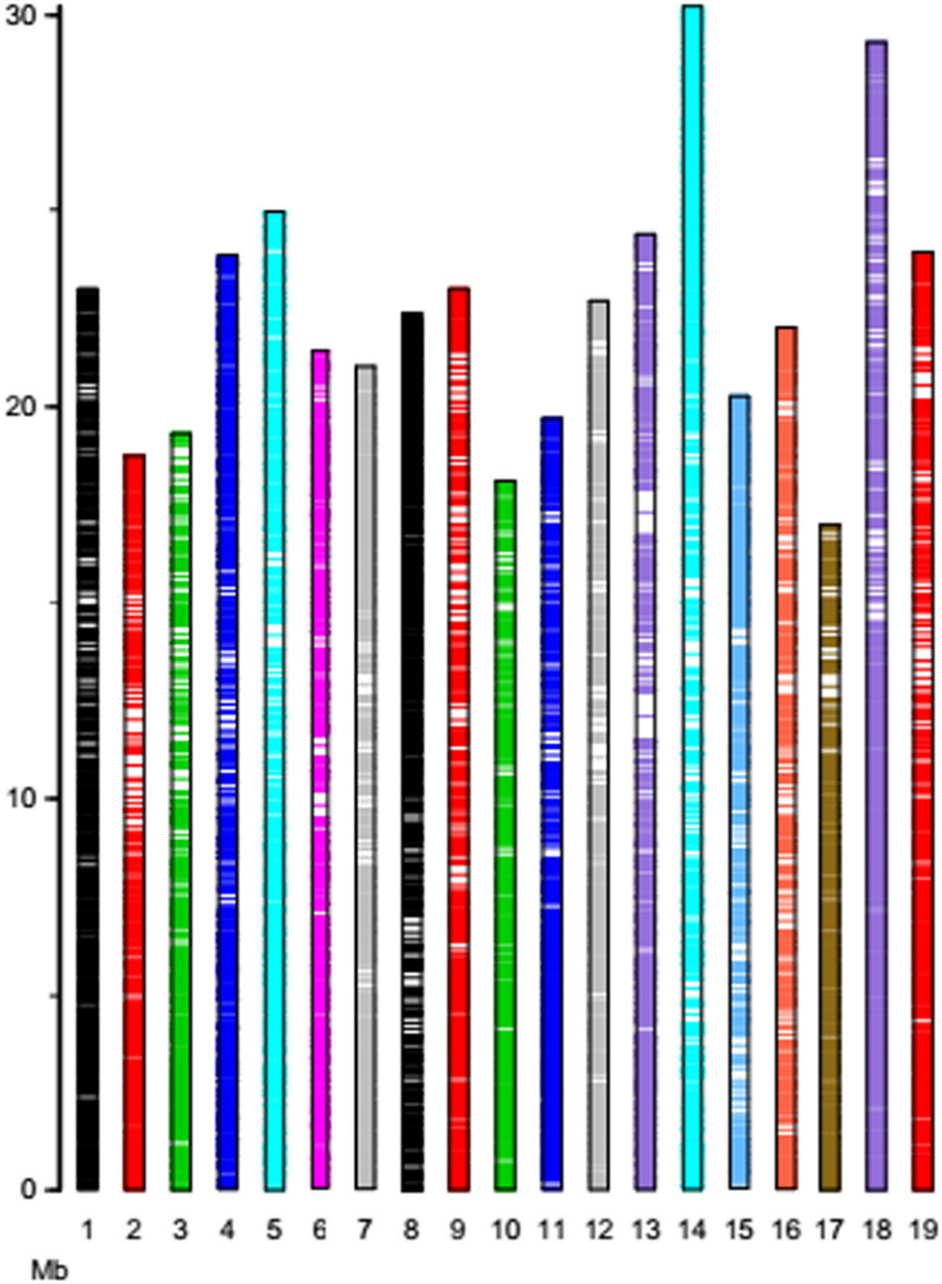
Single-nucleotide polymorphism (SNP) distributions on 19 chromosomes of grape. The vertical axis shows the chromosome length

A total of 43.71 GB of sequence data was generated for the 179 grape genotypes, including 864.45 million reads. The Q30 ratio and guanine–cytosine (GC) content were 92.08% and 45.14%, respectively. High-quality reads were aligned to the grape PN40024 genomic sequence. A total of 358,070 SNPs were initially obtained for these genotypes from the SOAPsnp utility calling. After removing those nucleotide polymorphisms with more than two alleles, a set of 306,015 SNPs was generated. After excluding those SNPs with an MAF of < 0.05 and a SNP detection rate of < 95%, 32,311 high-quality SNPs remained for further analysis. These high-quality SNPs covered all 19 chromosomes and were approximately evenly distributed across the whole genome (Fig. 1). The largest number of SNPs was found on chromosome 18 (2482 SNPs), followed by chromosome 14 (2359 SNPs), whereas the smallest number of SNPs was found on chromosome 17 (1059 SNPs). The distribution of SNPs on each chromosome was largely consistent with the physical length of the corresponding chromosome. The average marker density was ~14.26 kb/SNP. Chromosome 17 had the lowest SNP marker density (16.56 kb/SNP), and chromosome 8 had the highest marker density (11.84 kb/SNP).

### Genetic structure and linkage disequilibrium

**Fig. 2:**
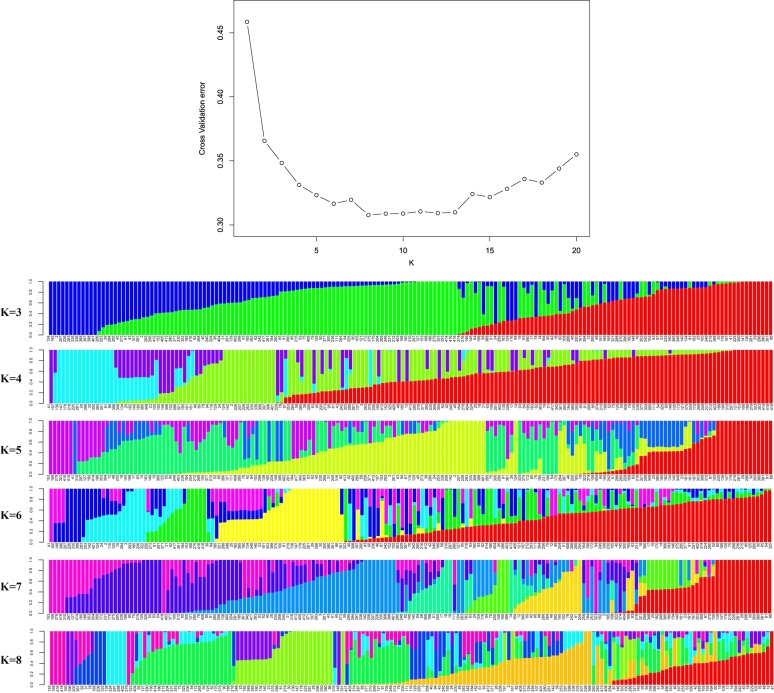
Population structure inferred by ADMIXTURE analysis. Top: Cross-validation plot for the SNP dataset. Bottom: Bar plots for K = 3–8. Each plot was created from 179 genotypes and is ordered by q values; each genotype is represented by a single vertical line, and each color represents one cluster

To better understand the genetic structure of the 179 grape genotypes, ADMIXTURE software was used to analyze the SNP data using the model-based, maximum-likelihood ancestry estimation procedure. The optimal number of inferred ancestral components (k) was estimated by ADMIXTURE’s cross-validation for the number of sub-populations (k), which varied from 2 to 20. Structure simulation demonstrated that the cross-validation error was minimized at k = 8 (Fig. 2). Hence, a k-value of 8 was selected to describe the genetic structure of the 179 grape genotypes.

**Fig. 3:**
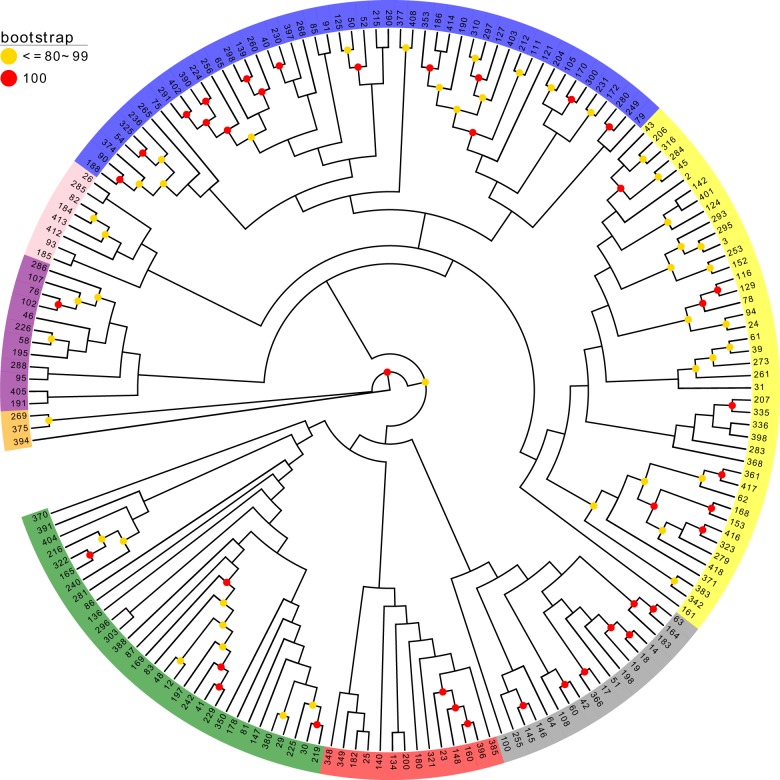
A dendrogram constructed by a neighbor joining (NJ) approach using information from 32,311 genome-wide SNPs. Operational taxonomic units (OTUs) are colored for different clusters according to the topology

To further assess genetic differentiation of the genotypes, neighbor joining (NJ) clustering of the samples was performed. Consistent with the ADMIXTURE results, the hierarchical clustering dendrogram showed distinct differentiation among the cultivar complexes (Fig. 3). Eight major clusters were defined in the dendrogram; the groupings corresponded to each of the major subgroups of the ADMIXTURE analysis, which supported the division of the population into eight major subgroups (Fig. 2). The corresponding Q-matrix at k = 8 was used for further marker–trait association mapping.

All 32,311 high-quality SNP markers were employed to estimate the LD extent in the association population. The average decay distance of LD was approximately 16.6 kb at the threshold of r^2^ = 0.03 across all chromosomes (Supplementary Fig. S2). The LD extent for a predicted r^2^ of 0.03 varied from 0.4 to 299 kb (Supplementary Fig. S2).

### Genome-wide association analysis

**Fig. 4:**
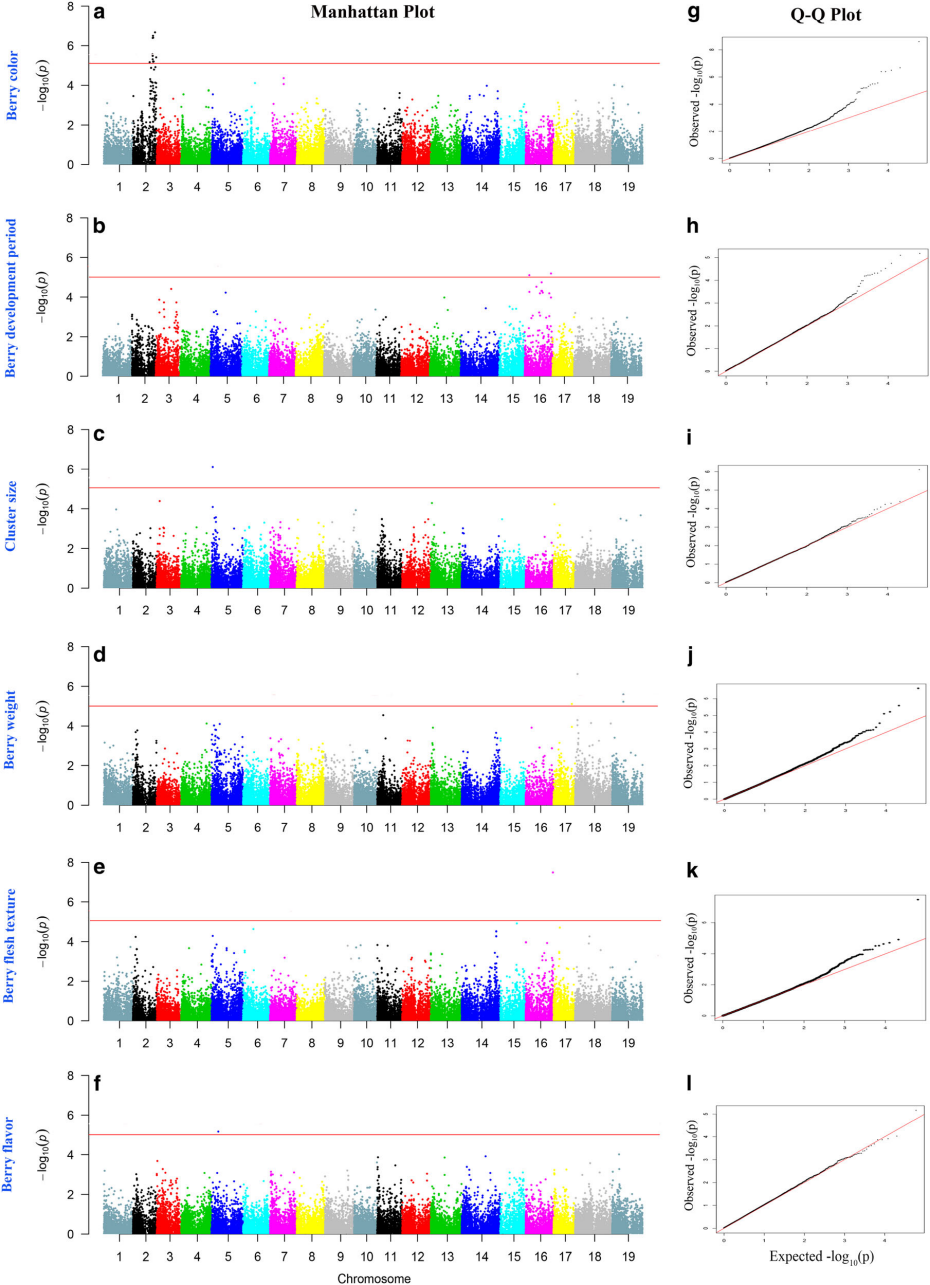
Manhattan plots of −log_10_(*P*) vs. chromosomal position (**a**–**f**) and Q–Q plots of SNP markers (**g**–**l**) from GEMMA models for berry color, berry development period, cluster size, berry weight, berry flesh texture, and berry flavor. The red horizontal line depicts the significance threshold (−log10 *P* = 5)

Association analyses of 32,311 SNP markers with eight berry traits of the 179 genotypes were performed to detect marker–trait associations using four different software programs: GEMMA^22^, EMMAX^21^, PLINK^25^, and TASSEL^20^. Quantile–quantile (Q–Q) plots of the observed and expected *P-*values were used to evaluate the fit of each model (Supplementary Fig. S1). A deviation from the identity line (*x* = *y*) was generally observed for the four models (Supplementary Fig. S1). Many tests yielded an associated *P-*value that was slightly higher than the expected *P-*value under the null hypothesis of no association, indicating associations for these traits. The GEMMA model had a better fit to the expected values than did other models for most of the eight traits (Supplementary Fig. S1); thus, the GEMMA model offered the best control of type I false positives. Therefore, only the association results obtained for the GEMMA model are shown and discussed here. Furthermore, no significant associations were detected for berry shape and cluster density. Figure 4 presents the Manhattan plots and Q–Q plots of the other six berry traits.

Application of the GEMMA model identified some SNPs significantly associated with six berry traits. An arbitrary number of top candidates were selected based on the probability value (*P* ≤ 0.001; P.adjust ≤ 0.005). The ~1 Mb regions of interest surrounding the significantly associated SNPs were scanned. In addition, the full regions between two associated SNPs were explored when GWAS detected specific patterns of association. The transcripts within or neighboring the associated loci were screened according to the grape genome of CRIBI (http://genomes.cribi.unipd.it/grape/).

### Berry color (BC)

Manhattan plots (Fig. 4) showed that a total of five SNP markers (Table 2) that were above the threshold for the GEMMA model were associated with BC and were all distributed on chromosome 2. The most significantly associated SNP marker was at chr2:14316046 (p = 2.56E-09), which

**Table 2:** Significant loci associated with grape berry traits as determined from GEMMA analysis and annotations of the candidate genes

Trait	Marker	*P*	P.adjust	Gene	Annotation	Region annotation^a^
Berry color	chr2:14316046	2.56E-09	1.19E-06	VIT_202s0033g00460	transcription factor mybA3	VIT_202s0033g00410 transcription factor myba3; VIT_202s0033g00450 transcription factor myba3
	chr2:15641010	4.18E-07	3.89E-05	VIT_202s0033g00880	60S ribosomal protein L8-3	
	chr2:15842558	3.25E-07	5.05E-05	VIT_202s0033g00960	nuclear-interacting partner of alk	
	chr2:15983296	4.07E-07	4.74E-05	VIT_202s0033g01000	benzyl alcohol o-benzoyltransferase	
	chr2:17439426	2.17E-07	5.06E-05	VIT_202s0087g00230	aspartic proteinase nepenthesin-1	
Berry development period	chr16:20841803	6.62E-06	2.15E-03	VIT_216s0098g00420	probable receptor-like protein kinase at1g67000-like	VIT_216s0098g00390 probable receptor-like protein kinase at1g67000-like; VIT_216s0098g00400 probable receptor-like protein kinase at1g67000-like; VIT_216s0098g00460 gdsl esterase lipase at4g10955; VIT_216s0098g00480 gdsl esterase lipase at4g10955
	chr16:3676395	7.97E-06	1.30E-03	VIT_216s0115g00110	myosin-like protein	VIT_216s0115g00170 seed maturation protein VIT_216s0115g00190 DNA-directed RNA polymerases and iii subunit rpabc4-like
Cluster size	chr5:1096673	7.86E-07	2.45E-02	VIT_205s0077g01350	AMSH-like ubiquitin thioesterase 3-like	
	chr5:1096614	8.33E-05	6.45 E-01	VIT_205s0077g01350	AMSH-like ubiquitin thiolesterase 3-like	
Berry weight	chr18:1954157	2.39E-07	9.94E-05	VIT_218s0001g01370	wall-associated receptor kinase 2-like	VIT_218s0001g01360 toprim domain-containing protein VIT_218s0001g01390 gibberellin 20-oxidase
	chr19:8859958	2.56E-06	5.30E-04	VIT_219s0015g00730	cellulose synthase-like protein e6-like	VIT_219s0015g00710 cellulose synthase-like protein e1 VIT_219s0015g00740 protein plastid movement impaired 2-like VIT_219s0015g00750 sucrase-like protein VIT_219s0015g00750 sec14 cytosolic factor-like
	chr19:8859967	6.02E-06	8.33E-04	VIT_219s0015g00730	cellulose synthase-like protein e6-like	
	chr17:14485590	7.81E-06	8.11E-04	VIT_217s0119g00330	uncharacterized protein	uncharacterized protein
Berry flesh texture	chr16:21539912	3.32E-08	1.24E-05	VIT_216s0098g01280	senescence-associated protein din1	VIT_216s0098g01140 calcium-dependent protein; VIT_216s0098g01180 aldo-keto reductase oxidoreductase; VIT_216s0098g01230 cytochrome b5; VIT_216s0098g01560 glucan endo-beta-glucosidase; VIT_216s0098g01590 calcium-binding EF-hand family protein
Berry flavor	chr5:5515972	6.95E-06	2.32E-03	VIT_205s0020g03860	homocysteine S-methyltransferase 2	VIT_205s0020g03640 c-8 sterol isomerase; VIT_205s0020g03710 gcn5-related n-acetyltransferase-like protein; VIT_205s0020g03930 sulfate bicarbonate oxalate exchanger and transporter sat-1

^a^The genes located in the regions were within approximately 1 Mb upstream or downstream of the SNPs of interest

corresponded to the transcripts of VIT_202s0033g00460 and were annotated as transcription factor *mybA3*. Further survey of the region around VIT_202s0033g00460 showed that VIT_202s0033g00430, VIT_202s0033g00440, and VIT_202s0033g00450 were all annotated as transcription factor *mybA3.*

### Berry development period (BDP)

A total of two SNP markers were associated with BDP above the threshold (Fig. 4), and two association signals were found on chromosome 16, which were distributed on two regions of chromosome 16. The transcript of VIT_216s0098g00420 was annotated as probable receptor-like protein kinase at1g67000-like and gdsl esterase lipase at4g10955 (Table 2). The second transcript of VIT_216s0115g00110 was annotated as seed maturation protein and DNA-directed RNA polymerases and iii subunit rpabc4-like (Table 2).

### Cluster size (CS)

Two SNP markers were identified as loci significantly associated with CS on chromosome 5 (Fig. 4, Table 2). However, as the P.adjust value was not significant (≤ 0.005), this trait was excluded from further analysis.

### Berry weight (BW)

A total of four loci exhibited significant associations with BW, which was distributed on chromosome 18, chromosome 19 and chromosome 17. The significantly associated loci on chromosome 18 were annotated as VIT_218s0001g01370 (wall-associated receptor kinase 2-like). The transcripts in this region included VIT_218s0001g01360 (toprim domain-containing protein) and VIT_218s0001g01390 (gibberellin 20-oxidase), whereas on chromosome 19, the two associated loci were annotated as VIT_219s0015g00730 (cellulose synthase-like protein e6-like). The genes that were found in this enlarged interval comprised VIT_219s0015g00710 (cellulose synthase-like protein e1), VIT_219s0015g00740 (protein plastid movement impaired 2-like), VIT_219s0015g00750 (sucrase-like protein), and VIT_219s0015g00750 (sec14 cytosolic factor-like). The transcripts of VIT_217s0119g00330 on chromosome 17 were listed as uncharacterized protein.

### Berry flesh texture (BFT)

Only one locus, VIT_216s0098g01280 (senescence-associated protein din1), was found to be significantly associated with BFT. VIT_216s0098g01140 (calcium-dependent protein), VIT_216s0098g01180 (aldo-keto reductase oxidoreductase), VIT_216s0098g01230 (cytochrome b5), VIT_216s0098g01560 (glucan endo- -beta-glucosidase), and VIT_216s0098g01590 (calcium-binding EF-hand family protein) were within or neighboring the locus (Table 2).

### Berry flavor (BF)

Manhattan plots (Fig. 4) and GEMMA analysis (Table 2) showed that one SNP on chromosome 5, VIT_205s0020g03860 (homocysteine S-methyltransferase 2), was significantly associated with BF. Moreover, most of the transcripts around the locus were related to substance metabolism, including VIT_205s0020g03640 (c-8 sterol isomerase), VIT_205s0020g03170 (gcn5-related n-acetyltransferase-like protein), and VIT_205s0020g03930 (sulfate bicarbonate oxalate exchanger and transporter sat-1).

## Discussion

### GWAS population

GWAS is a powerful strategy that uses natural populations to understand the genetic bases of complex traits. This strategy employs historic LD to link phenotypes to genotypes and thereby predict marker–trait associations^12^. GWAS has been widely used for mapping important traits in diverse plants^9^. A large number of diverse accessions need to be covered to accurately capture the genome-wide distribution of LD and to identify markers linked to various traits. In this study, we chose 179 grape genotypes originating from different countries (Supplementary Table S1) to obtain an association panel with high genetic diversity. In practice, successful GWAS analyses have been reported for 107 natural accessions of *Arabidopsis thaliana*^27^, but most studies used accession sizes between 100 and 500^28^. PCA was performed to quantify the population structure of these 179 genotypes (Supplementary Fig. S3) based on nucleotide polymorphisms. There were no distinct clusters presented in the plot of principal components, indicating that the genotypes employed did not represent a highly structured population, according to Yano et al^29^.

Several association studies have been conducted in grape for the characterization of genetic variation, population structure, and LD using SSR and SNP markers^8,14,16,17,30^. These studies have shown that LD between SNPs in grape may vary widely across and within species depending on the genotypes represented in the diversity panels. LD was found to decay faster within the domesticated grape than in its wild relative; LD (r^2^) decayed below 0.2 within 10 kb^31^. In other grape accession panels, LD reached 0.2 at physical distances of ca. 250 bp^30^ and < 10 kb^14^. In the present study, pairwise LD analysis revealed that LD reached the threshold (r^2^ = 0.03) at an average physical distance of 16.6 kb and a range of distances from 0.4 to 299 kb (Supplementary Fig. S2). The LD decay using genome-wide SNPs in domesticated grape in this study was comparable to that of Myles et al^14^. and Marrano et al^31^. under the same model of bin medians.

The loci on LG2 responsible for the presence or absence of skin color were identified in grape segregating mapping populations^1,2^ and colocalized with a cluster of *VvMYB* genes (*VvmybA1*, *VvmybA2*, *Vvmyb3*, and *Vvmyb4*)^7^. Because the genes responsible for berry color are well known^32^, we selected the trait of berry color as a reference trait to evaluate the efficiency of the association models. An anthocyanin-related clade of a group of nine R2R3–MYB genes were identified within a 150 kb (14.16-14.31 M) cluster on chromosome 2^33^. Based on the population in this study, we successfully identified significant loci associated with grape berry color, which were in the region of chr2:14316046 (around 14.31 M) (Fig. 4) and annotated as transcription factor *mybA3* (Table 2). This result is consistent with previous reports and indicates that the population we employed in this study was suitable for conducting association mapping.

### Selection of statistical models

The selection of optimal statistical models is beneficial for accurately evaluating the associations between markers and phenotypes. As genotypic data become increasingly available, more comprehensive statistical models are needed to distinguish true biological associations from false positives that result from population structure and LD^34^. Numerous statistical models are available to calculate the significance of associations between SNPs and traits^28^. Yu et al^35^. developed a method based on a mixed linear model (MLM) that can correct for population structure and family relatedness. There are many offline and online software programs that run GWAS analysis based on general linear models (GLMs) and MLMs^28^, such as PLINK^25^ and TASSEL;^20^ these programs are commonly used for association analyses in plants. However, the definitions of unrelated individuals suggested by Yu et al^35^. are somewhat arbitrary; and for the MLM approach, the computational demand is heavy for datasets with large samples^20^. Improvement in EMMAX^21^ was accomplished through complex approximate estimation methods for variance components that reduced computational processing^34^. However, it is difficult to predict the accuracy of variance components without running an exact calculation for EMMAX. GEMMA presents the advantage that it directly and accurately estimates variance components, including fixed effects in MLM, which reduces the calculation burden^22^.

In this study, four statistical models of GWAS analysis–PLINK^25^, TASSEL^20^, EMMAX^21^, and GEMMA^22^–were used to predict loci significantly associated with grape berry quantitative traits.

A Q–Q plot is a graphic representation of the deviation of observed *P*-values from expected *P*-values. The Q–Q plots of eight berry traits from four statistical models are shown in Supplementary Fig. S1. In general, the results from PLINK and TASSEL showed that the observed *P*-values deviated from the expected values, indicating that these two models were not appropriate for this study. The Q–Q plot results from GEMMA and EMMAX showed high associations in eight berry traits. The GEMMA model showed better fit to the expected values than did the EMMAX model and therefore offered better control of type I false positives. Regarding the berry color trait, the GEMMA method presented substantially higher power for significant loci detection than did the EMMAX method, regardless of what *P*-value criteria were used; and the loci detected were consistent with previous reports. Consequently, only the association results obtained from GEMMA are shown and discussed for the other berry traits.

### Potential candidate genes for berry traits

We evaluated the GWAS results in light of previous reports and functional information. The most intriguing result of the current study was that many of the QTLs identified based on the GWAS were in accordance with the results of previous QTL analyses.

The grape color locus has been identified as being a cluster of *MYB*-type transcription factor genes on chromosome 2^33^, which has been verified by other reports^7^. The position of the significantly associated SNPs detected in this study was annotated as *mybA3* and around 14.31 Mb on chromosome 2, which is not the exact position of the known causal mutation sites of the grape color locus (*VvMybA1 and VvMybA2*). A similar result was found in the association mapping analysis of Migicovsky et al^17^., who found that the SNPs significantly associated with skin color were 3.6 Mb from the known causal mutation site on chromosome 2. Similarly, the strongest association signals detected by GWAS in rice^36^ and *Arabidopsis*^27^ were not at the exact known loci of the target traits. Previous studies have proved that mutation of two functional *MYB*-related genes (*mybA1* and *mybA2*) result in white-skinned cultivars^33^, whereas *mybA3* is regarded as non-functional^32^. Azuma et al^37^. reported that a functional *VvmybA1* in ‘Benitaka’ (*V. vinifera* L.) was restored from homologous recombination between *VvmybA1a* and *VvmybA3*. Many *MYB*-like genes distributed in the region of *VvmybA1* to *VvmybA3* might be associated with the different colors of grape berry skins and are not necessarily functional^37^*. VvmybA3* has been detected in cultivars of *V. vinifera* and its hybrids^38^. The above observations support the annotation of the identified SNP loci associated with BC as *mybA3* in this study.

BDP is a trait that is dependent on environmental conditions; however, different varieties have different development periods. Thus, there might be a specific genetic control for this trait regardless of environmental conditions^3^. A QTL affecting veraison on chromosome 16 was identified by Costantini et al^4^. In addition, six independent quantitative trait loci were detected for veraison on chromosomes 16 and 18, and a QTL associated with veraison on chromosome 16 around marker VVMD37 was identified^39^. However, the position of this trait differs between previous studies and Duchêne et al^39^., although all are located on chromosome 16. CMa3, one of the QTLs related to grape maturation period, was detected on chromosome 16 near the marker UDV052 based on a hybrid (F1) population in which an early-maturing female parent “87-1” was crossed with a late-maturing male parent ‘9-22’^40^. Zyprian et al^24^. identified a prominent QTL on chromosome 16 associated with the timing of the onset of veraison near the marker of UD0V52. In the present study, two SNPs considered to have the strongest associations with BDP were both on chromosome 16. The genes around the significantly associated SNP locus (VIT_216s0098g00420) were annotated as probable receptor-like protein kinase at1g67000-like. Zhang et al^41^. and Jia et al^42^. revealed the important role of receptor kinase during fruit ripening in strawberry and apple, respectively. These results suggest that the association signals revealed by this study are reliable. Moreover, control of the BDP trait in grape may be complicated, and the candidate genes detected in this study are worthy of further investigation.

Previously published QTL studies in seedless grape demonstrated that berry weight is controlled by a major QTL related to seed traits on chromosome 18 near the SSR marker VMC7F2^43^. Since berry weight and berry size (diameter) are highly correlated, overall berry size can be analyzed with either trait^3^. Seed development inhibitor (SDI) has been detected on chromosome 18 by different authors in different years and for different progenies^7^. The GWAS results of this study confirm the existence of significantly associated SNPs for berry weight on chromosome 18. However, the position of the loci in this study differed from that determined from biparental QTL mapping. This discrepancy may be due to the previous studies’ employment of segregating populations derived from crosses involving seedless cultivars, in which the SNPs colocalized with QTLs of seed number, indirectly affecting berry size^44^. Chialva et al^44^. identified the transcript of VIT_218s0001g08610, annotated as VviANT1 (AINTEGUMENTA), as the transcript of a candidate gene of berry size (berry weight). The gene for berry weight identified from the GWAS analysis in the present study was VIT_218s0001g01370, near VviANT1. Further investigation of this associated region showed that the annotated genes of wall-associated receptor kinase 2-like and gibberellin 20-oxidase were located there. Costantini et al.^4^ also localized a marker of berry weight corresponding to gibberellin 20-oxidase. Thus, we verified the relationship of the gibberellin 20-oxidase gene with grape berry weight. Another region associated with berry weight detected in this study was located on chromosome 19 and chromosome 17. Previous reports have not described the corresponding QTLs in these regions. Berry weight is affected by numerous factors (cell wall modification, cell multiplication, photosynthesis, growth regulators, etc.) and is expected to be controlled by polygenes, with different causal polymorphisms segregating in different populations^43^. The functional roles of these regions are unclear. More genetic and molecular evidence will be needed to confirm this association.

Berry flesh texture, i.e., berry firmness, is an important quality trait in table grape production^6^. However, its genetic determination is poorly understood. A complex genetic control of grape firmness was revealed by QTL analyses. Carreño et al.^45^ detected QTLs for berry firmness at the genome-wide level in seven genomic regions on chromosomes 1, 4, 5, 9, 10, 13, and 18.

Two QTLs for firmness were found to be located on chromosome 3 and chromosome 10 by Ban et al.^5^ Correa et al.^6^ showed that the determinants for this trait are distributed in chromosome 8 and chromosome 18. Previous QTL studies revealed polygenic control of this trait. In the present study, we mapped the SNPs for this trait on chromosome 16 by GWAS analysis. One explanation for this lack of agreement among studies is that this trait might be controlled by many genes involved in complex metabolic pathways^45^. Among the tens of genes found in the two QTLs of Correa et al^6^., a cation/calcium exchanger gene was highlighted. Among the genes found for this SNP of chromosome 16 in the present study, two genes were related to calcium: VIT_216s0098g01140 (calcium-dependent protein) and VIT_216s0098g01590 (calcium-binding EF-hand family protein). Calcium is an essential nutrient with structural roles in the cell wall, and it has an important impact on fruit firmness by reducing the action of cell wall-degrading enzymes^46^. Balic et al.^47^ demonstrated that varieties with lower calcium content at harvest were less firmer than those with higher calcium concentration in the cell wall. The functions of other genes need to be analyzed. One of the factors explaining the lack of stability of these QTLs is the high sensitivity of this trait to environmental changes or planting practices.

Berry flavor depends on the aromas of grape, which arise from volatile compounds, such as terpenes, norisoprenoids, and thiols. Linalool, geraniol, nerol, citronellol, and α-terpineol are generally regarded as the main monoterpenes in Muscat cultivars^48^. In this study, berry flavor was associated with chr5:5515972. These marker–trait associations corroborate previous QTL mapping and association analysis studies in grape that identified similar genic regions. Muscat flavor has been determined by evaluating monoterpenoid levels through QTL analysis in grape. Doligez et al.^49^ described the QTL for muscat flavor on chromosome 5 based on tasting data. Battilana et al.^50^ identified 1-deoxy-d-xylulose-5-phosphate synthase (VvDXS) on chromosome 5 as a candidate gene for geraniol, nerol, and linalool content. Emanuelli et al^19^. demonstrated co-localization of 1-deoxy-D-xylulose 5-phosphate synthase (VvDXS) with the major QTL positioned on chromosome 5. Yang et al.^18^ found that DXS was nearest to UDV060 (4.4 Mb) on chromosome 5 based on GWAS analysis, a finding also obtained by Doligez et al^49^. Thus, both the association mapping approach and QTL analyses have mapped the QTL for berry flavor at the identified region and indicate that a major QTL is located on chromosome 5.

Ultimately, this study advances our understanding of the genetic control of grape berry traits and provides insight into the genetic control of grape berry traits. Our work provides new evidence that may lead to new research areas in grape molecular breeding.

## Electronic supplementary material

Supplementary Figure S1: QQ-plot for eight berry traits of grape resulted from the different statistical models

Supplementary Fig.S2: Decay of linkage disequilibrium with genome distance in grape

Supplementary Fig.S3: Two-dimensional principal component analysis (PCA) plot of the 179 genotypes. PCA analysis was based on the SNP data from the GBS

Supplementary Table S1: Phenotypic description of 179 grapegenotypes used in this study
