# Supplementary material for: Genome-wide association study of berry-related traits in grape [Vitis vinifera L.] based on genotyping-by-sequencing markers
Source: Hortic Res. 2019 Jan 1;6:11. doi: 10.1038/s41438-018-0089-z (PMC6312537; doi:10.1038/s41438-018-0089-z)
Supplement: Supplementary file 1 — Supplementary Figure S1: QQ-plot for eight berry traits of grape resulted from the different statistical models [file 41438_2018_89_MOESM1_ESM.doc]

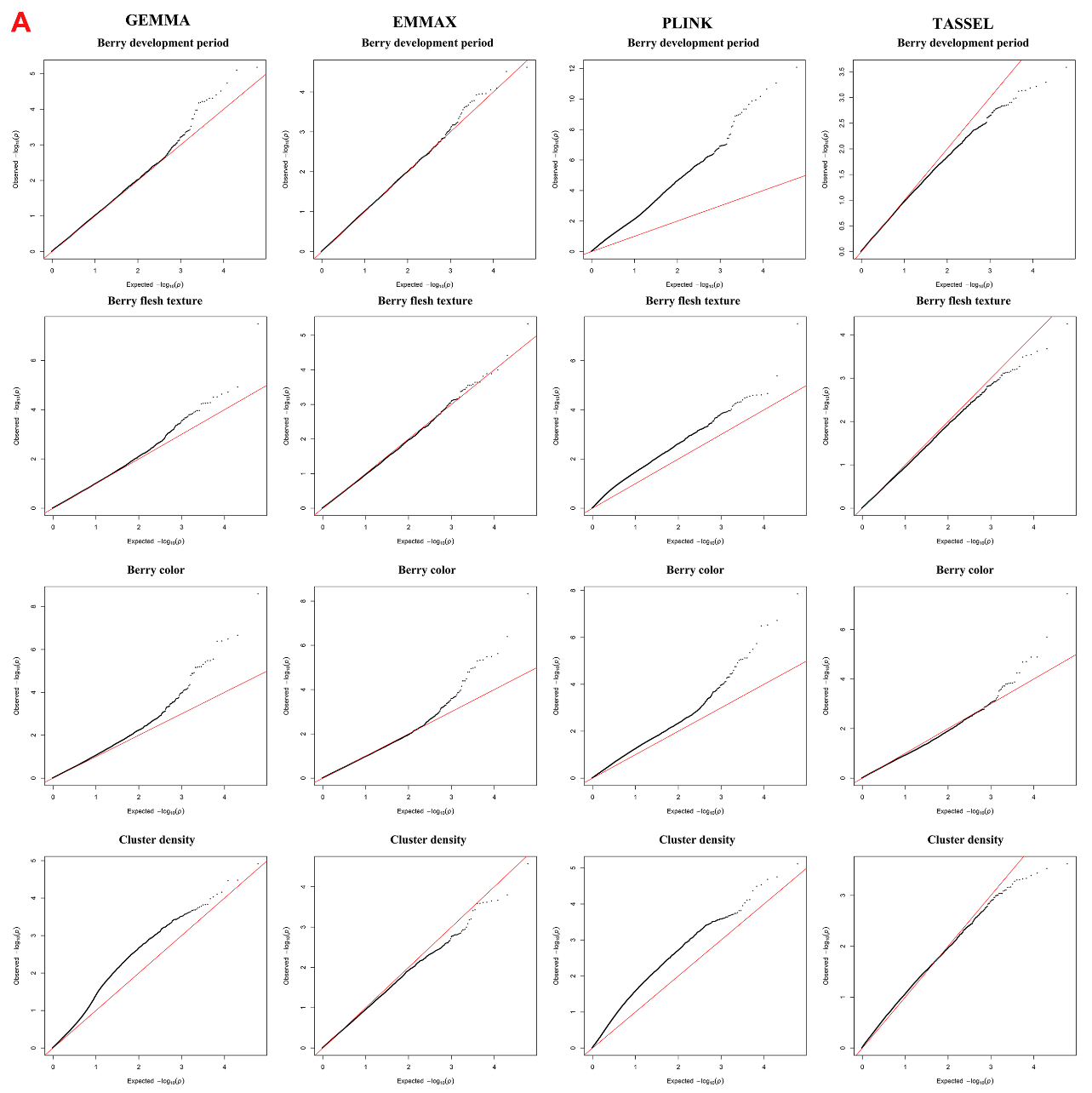


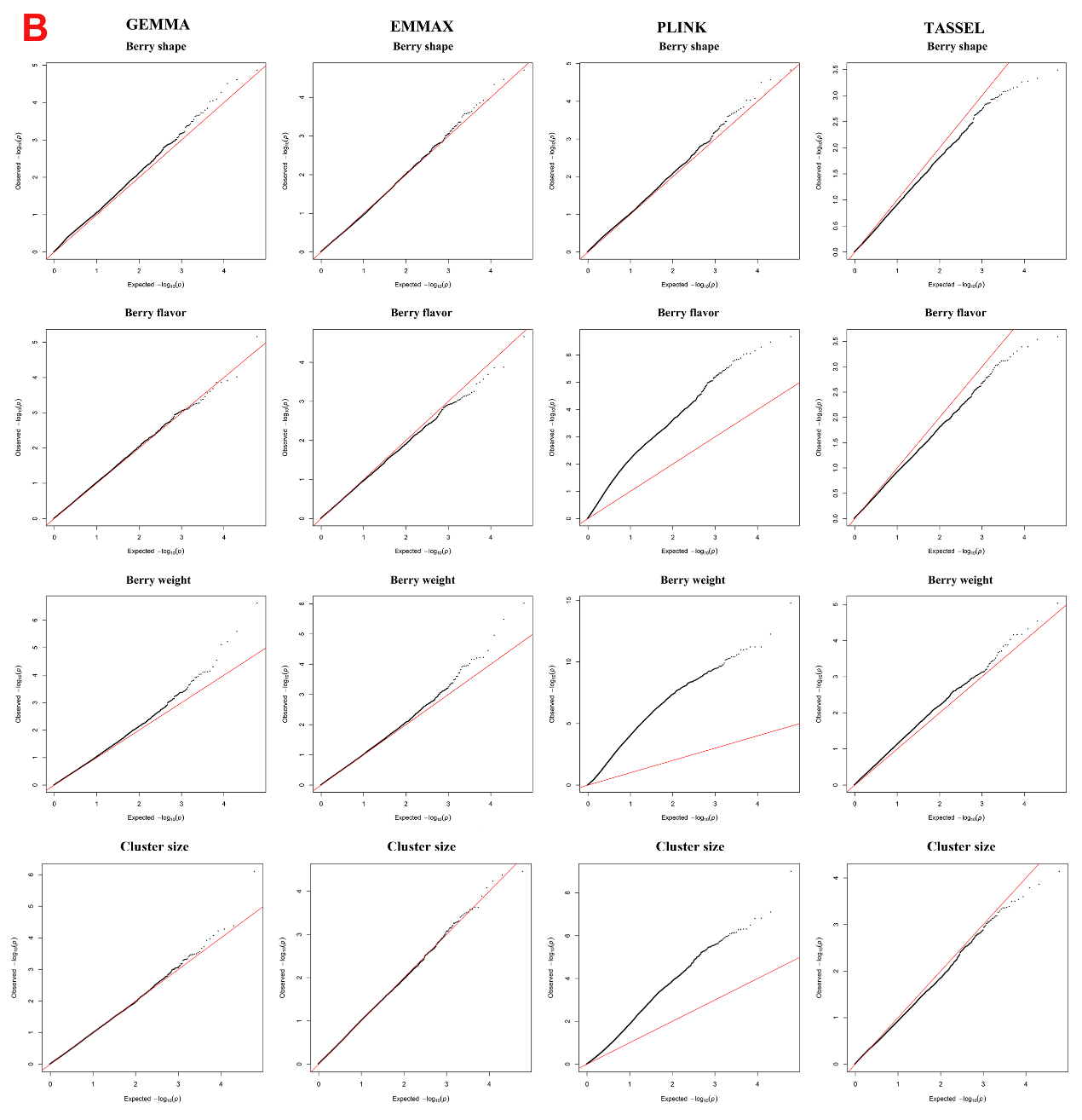


Supplementary Figure S1: QQ-plot for eight berry traits of grape resulted from the different statistical models. X-axis shows expected –log(p-value); y-axis shows observed –log(p-value). From left to right: GEMMA, EMMAX, PLINK, TASSEL.

A showed QQ-plot of berry development period, berry flesh texture, berry color and cluster density;

B showed QQ-plot of berry shape, berry flavor, berry weight and cluster size.
