# Supplementary figures and images for: Genome-wide association study of berry-related traits in grape [Vitis vinifera L.] based on genotyping-by-sequencing markers
Source: Hortic Res. 2019 Jan 1;6:11. doi: 10.1038/s41438-018-0089-z (PMC6312537; doi:10.1038/s41438-018-0089-z)

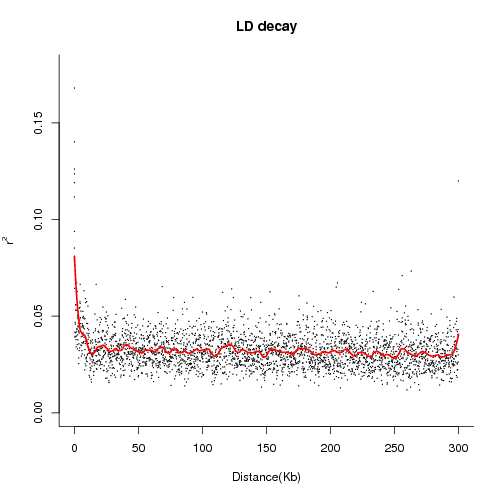


Supplementary Fig.S2: Decay of linkage disequilibrium with genome distance in grape.

Supplement: Supplementary file 2 — Supplementary Fig.S2: Decay of linkage disequilibrium with genome distance in grape [file 41438_2018_89_MOESM2_ESM.doc]
