# Supplementary material for: Genome-wide association study of berry-related traits in grape [Vitis vinifera L.] based on genotyping-by-sequencing markers
Source: Hortic Res. 2019 Jan 1;6:11. doi: 10.1038/s41438-018-0089-z (PMC6312537; doi:10.1038/s41438-018-0089-z)
Supplement: Supplementary file 3 — Supplementary Fig.S3: Two-dimensional principal component analysis (PCA) plot of the 179 genotypes. PCA analysis was based on the SNP data from the GBS [file 41438_2018_89_MOESM3_ESM.doc]

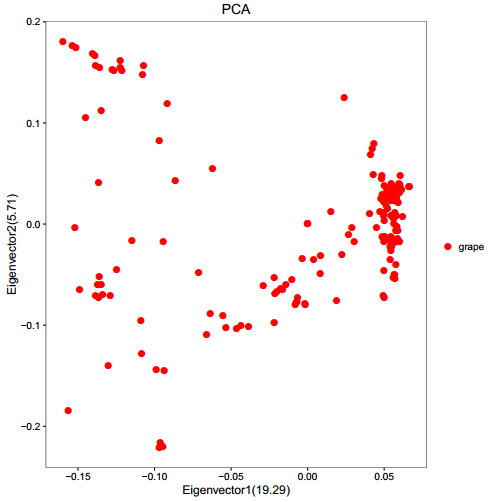


Supplementary Fig.S3: Two-dimensional principal component analysis (PCA) plot of the 179 genotypes. PCA analysis was based on the SNP data from the GBS.
